# Nanofibrous Microspheres: A Biomimetic Platform for Bone Tissue Regeneration

**DOI:** 10.1021/acsabm.4c00613

**Published:** 2024-07-01

**Authors:** Nimeet Desai, Shreya Pande, Lalitkumar K. Vora, Nagavendra Kommineni

**Affiliations:** † Department of Biomedical Engineering, Indian Institute of Technology Hyderabad, Kandi 502285, India; ‡ School of Pharmacy, Queen’s University Belfast, 97 Lisburn Road, Belfast BT9 7BL, United Kingdom; § Center for Biomedical Research, Population Council, New York, New York 10065, United States

**Keywords:** Microspheres, Biomimetic, Bone regeneration, Tissue engineering, Scaffold

## Abstract

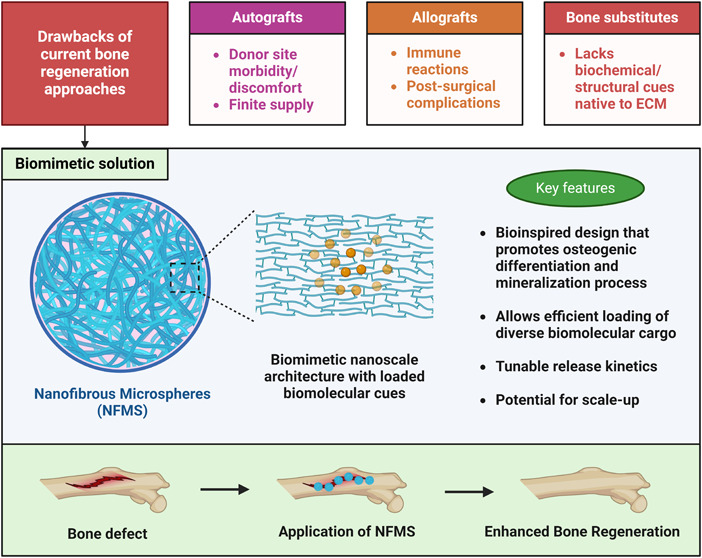

Bone, a fundamental constituent of the human body, is a vital scaffold for support, protection, and locomotion, underscoring its pivotal role in maintaining skeletal integrity and overall functionality. However, factors such as trauma, disease, or aging can compromise bone structure, necessitating effective strategies for regeneration. Traditional approaches often lack biomimetic environments conducive to efficient tissue repair. Nanofibrous microspheres (NFMS) present a promising biomimetic platform for bone regeneration by mimicking the native extracellular matrix architecture. Through optimized fabrication techniques and the incorporation of active biomolecular components, NFMS can precisely replicate the nanostructure and biochemical cues essential for osteogenesis promotion. Furthermore, NFMS exhibit versatile properties, including tunable morphology, mechanical strength, and controlled release kinetics, augmenting their suitability for tailored bone tissue engineering applications. NFMS enhance cell recruitment, attachment, and proliferation, while promoting osteogenic differentiation and mineralization, thereby accelerating bone healing. This review highlights the pivotal role of NFMS in bone tissue engineering, elucidating their design principles and key attributes. By examining recent preclinical applications, we assess their current clinical status and discuss critical considerations for potential clinical translation. This review offers crucial insights for researchers at the intersection of biomaterials and tissue engineering, highlighting developments in this expanding field.

## 1 Introduction

Bone, as a fundamental component of the human body, plays multifaceted roles essential for maintaining physiological homeostasis. Structurally, it provides support and protection and facilitates locomotion, thus underlining its indispensable function in ensuring overall bodily function.^1^ Moreover, bone serves as a reservoir for crucial minerals, such as calcium and phosphorus, which are pivotal for various metabolic processes. Additionally, it acts as a dynamic tissue, continuously undergoing remodeling through the coordinated activity of osteoblasts and osteoclasts, a process vital for maintaining bone strength and integrity.^2^ Despite its remarkable regenerative capacity, bone is susceptible to damage and degeneration due to an array of factors. Traumatic injuries, such as fractures or extensive bone loss resulting from accidents or sports injuries, often necessitate interventions to facilitate proper healing and restoration of function. Moreover, degenerative conditions like osteoporosis, osteoarthritis, and bone infections can compromise bone integrity, leading to pain, disability, and decreased quality of life.^3^

Traditionally, the management of bone defects and fractures has relied on surgical interventions coupled with bone grafting techniques. Autografts, harvested from the patient’s own body, have been considered the gold standard due to their osteogenic potential and low risk of immune rejection. However, autograft procedures pose challenges, including limited graft availability, donor site morbidity, and additional surgical risks.^4^ Allografts, derived from cadaveric sources, offer an alternative but are associated with concerns regarding immune compatibility and disease transmission.^5^ Furthermore, synthetic bone substitutes, while readily available, often lack the biological cues necessary for optimal tissue integration and regeneration.^6^ In response to these challenges, there has been a paradigm shift toward developing biomimetic approaches for bone tissue engineering.^7^ These approaches aim to replicate the complex microenvironment of native bone tissue by employing biomaterials that mimic the composition, structure, and functionality of the extracellular matrix (ECM).

**Figure 1:**
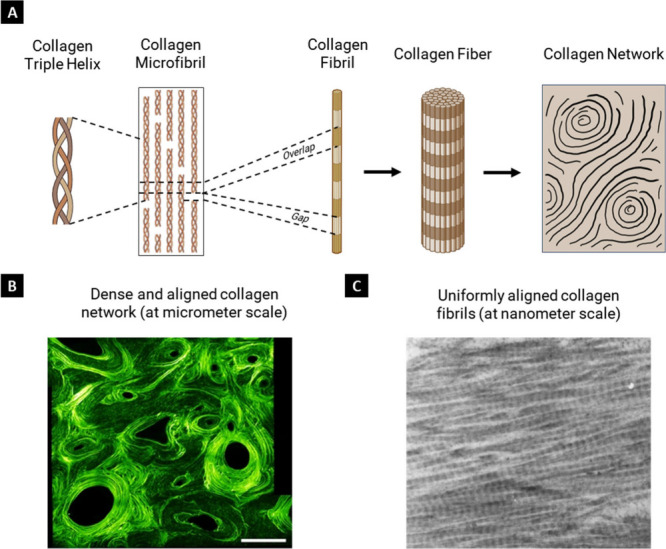
(A) Assembly of collagen from its fundamental structures to a complex network that forms the ECM within the bone. The organic matrix of bone is characterized by organized collagen fibrils at the nanometer scale and a densely aligned collagen fiber network at the micrometer scale. (B) Second harmonic generation image depicting a densely packed and aligned collagen fiber network in human femoral cortical bone. This image vividly captures the robust and organized texture of collagen fibers within the network, highlighting their alignment and density, essential for the mechanical strength of bone (scale bar, 200 μm). Reproduced with permission from ref (17). Copyright 2017 Springer Nature. (C) Electron microscopy image of cortical bone collagen fibrils. This high-resolution image provides a detailed view of the parallel alignment of individual collagen fibrils, underscoring the precision of molecular organization at the smallest scale within bone ECM. Reproduced with permission from ref (18). Copyright 2000 Elsevier.

The bone ECM is composed of approximately a 30–40% organic matrix, which imparts both toughness and elasticity to the bone.^8^ This matrix primarily consists of type I collagen produced by osteoblasts. These collagen molecules form triple helices that align into microfibrils in a twisted, staggered pattern, creating a distinctive gap between each molecule. These microfibrils aggregate into larger collagen fibrils, which further bundle to form collagen fibers. The arrangement of these fibers plays a crucial role in bone’s mechanical strength.^9^ Specifically, fibers aligned parallel to the direction of the load exhibit greater resistance to tension, while those perpendicular are more resistant to compression.^10^ The density and organization of the collagen network are critical for the adequate mineralization of bone.^11^ Bone structure can vary, ranging from immature woven bone, characterized by a chaotic arrangement of loosely packed fibrils, to mature lamellar bone, which features densely packed collagen fibers aligned parallel within each lamella but with alternating orientations across lamellae.^12^ Lamellar bone develops during the bone remodeling process, where osteoblasts align in a polarized manner along a surface, depositing collagen fibrils in a parallel orientation.^13^ Besides collagen, a smaller fraction of the organic matrix includes noncollagenous proteins (NCPs), which are crucial for the assembly of collagen fibrils and their subsequent mineralization. This combination of the collagen network, the organization of individual collagen fibrils, and NCPs serve as a foundational template for mineralization, significantly influencing the final structure of the ECM.^14^ Hydroxyapatite (Ca_5_(PO_4_)_3_OH) is the primary inorganic component of bone and forms through biomineralization. This process is regulated by the interactions between minerals and the bone matrix, particularly the amino acids in noncollagenous proteins, which guide the formation of hydroxyapatite.^15^ Collagen, produced during tissue mineralization, serves as a base scaffold for hydroxyapatite deposition. Hydroxyapatite’s chemical and physical properties closely resemble those of human bone minerals, making it biocompatible and osteoconductive.^16^Figure 1 provides a schematic illustration of the structural hierarchy and organization of collagen that constitutes the ECM of bone.

By providing a bioactive scaffold that closely resembles the natural bone microenvironment, biomimetic strategies hold immense potential for enhancing bone regeneration and overcoming the limitations of traditional approaches.^19^ Within this landscape, nanofibrous microspheres (NFMS) have emerged as a promising biomimetic platform for bone tissue regeneration. These microspheres offer a high surface area-to-volume ratio and can be precisely engineered to mimic the nanostructure and biochemical cues of the native ECM.^20,21^ Through their ability to support cell adhesion, proliferation, and differentiation, as well as facilitate the controlled release of bioactive molecules, NFMS present a versatile and effective approach for promoting bone healing.^22^ This work presents the first comprehensive review of NFMS specifically for bone tissue regeneration, an area previously unexplored in such depth. It highlights recent developments in NFMS technology, from design principles and cutting-edge fabrication methods to the critical features that confer biomimetic properties. By consolidating the latest preclinical studies and addressing translational considerations like market potential and regulatory challenges, this review uniquely positions NFMS at the forefront of biomaterials research in the context of bone tissue engineering.

## 2 Need of Biomimetic NFMS

NFMS represent innovative three-dimensional biomaterial constructs featuring nanoscale fibers (50–500 nm) organized into spherical microstructures. The external topological configuration of these microspheres is meticulously engineered to mimic the complex architecture observed in the ECM inherent to biological tissues.^23^ NFMS hold considerable promise, particularly in the realms of tissue engineering and regenerative medicine, owing to their ability to closely emulate native tissue environments.

The ECM forms a complex network of macromolecules that surrounds cells in tissues and organs across the body. In bone tissue, the ECM forms a dynamic and highly specialized environment that plays essential roles in maintaining skeletal structure, regulating cellular behavior, and facilitating tissue remodeling. Comprising an assorted combination of proteins, proteoglycans, and minerals, the ECM serves dual roles by providing both mechanical support and biochemical signaling cues vital for bone development, maintenance, and regeneration.^24^ Collagen fibrils and mineralized matrix are pivotal constituents conferring tensile and compressive strength to bone, respectively, thereby ensuring its resilience against deformation. In close association with the ECM, various bone cells, including osteoblasts, osteocytes, and osteoclasts, find attachment sites, facilitating their adhesion, migration, and intercellular communication.^25^ Notably, these cell–ECM interactions intricately regulate fundamental cellular processes such as proliferation, differentiation, and matrix synthesis, which are imperative for maintaining bone homeostasis and facilitating repair.^26^ Central to the dynamic process of bone remodeling are the orchestrated activities of osteoblasts and osteoclasts, both intimately guided by biochemical signals embedded within the ECM.^27,28^ These signals, encompassing growth factors, cytokines, and signaling molecules, modulate the functions of osteoblasts and osteoclasts, thereby regulating bone resorption and formation (Figure 1A). This orchestrated interplay ensures the maintenance of skeletal integrity and the adaptation of bone tissue to changing mechanical demands.^29^

Biomimetic materials constitute synthetic substances meticulously engineered to replicate the structure, functionality, and characteristics of natural biological materials prevalent in living organisms.^30,31^ Rooted in the concept of biomimicry or bioinspiration, this approach harnesses insights from nature’s design principles to develop novel materials and technologies mirroring the efficiency and effectiveness observed in biological systems.^32^ For successful bone tissue regeneration, it is crucial to mimic the native ECM within the design of biomaterials. Such biomimetic scaffolds aim to recreate microenvironments that authentically mimic the biochemical and mechanical cues pivotal for successful bone tissue regeneration. Researchers aim to improve bone regeneration therapies by developing scaffolds that mimic the native tissue environment. This approach enhances cell–material interactions, promotes tissue integration, and increases the overall effectiveness of the treatments.^33^ An ideal biomimetic scaffold should not only mimic the essential characteristics of the ECM but also support tissue remodeling. It should integrate effortlessly with the surrounding host tissue after serving its therapeutic purpose, ensuring long-term stability and functionality of the regenerated bone.^34^ Moreover, biomaterials endowed with immunomodulatory properties similar to those of the native ECM hold promise in regulating inflammation, fostering tissue healing, and mitigating adverse immune responses, thereby improving the overall success of bone regeneration therapies.

NFMS stands out as an exemplary biomimetic scaffold owing to its distinctive structural and physiochemical attributes. Diverse fabrication techniques afford precise manipulation of NFMS morphology and mechanical properties, encompassing parameters such as porosity, pore size, and stiffness.^35^ This inherent tunability facilitates tailoring NFMS to match the mechanical demands inherent to diverse bone defects and tissues, thereby ensuring optimal support and seamless integration. Moreover, NFMS can be ingeniously engineered to encapsulate and deliver bioactive molecules in a controlled and sustained manner. This controlled release mechanism enables meticulous modulation of cellular behavior and tissue regeneration processes, thereby amplifying therapeutic efficacy while mitigating potential side effects.^36^ Notably, NFMS can be engineered to exhibit stimuli-responsive behavior, wherein their properties undergo change in response to external stimuli like pH, temperature, or mechanical forces. Exploiting this responsiveness allows researchers to achieve on-demand release of bioactive molecules, thereby further augmenting their therapeutic potential.^37^

**Figure 2:**
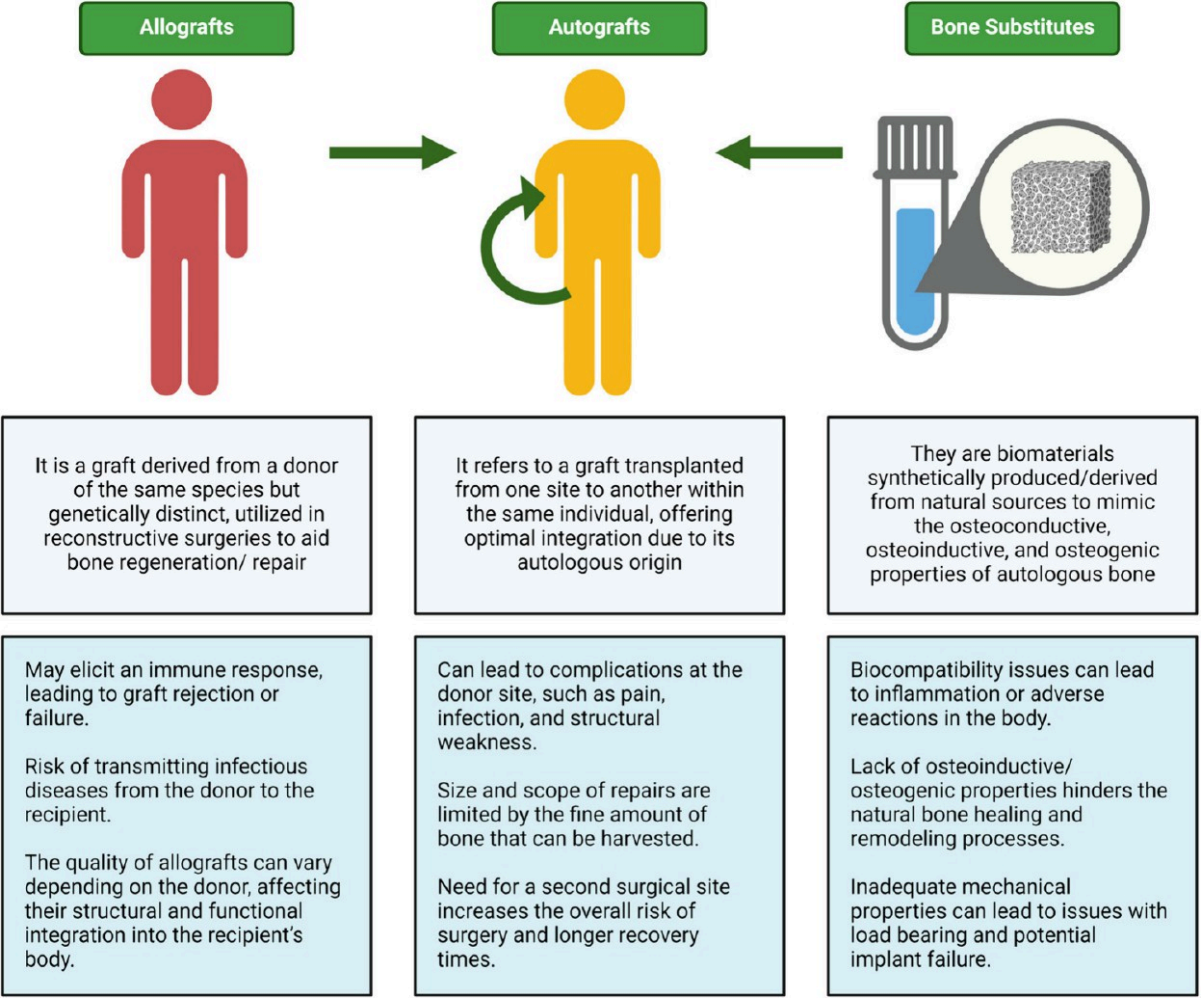
Comparative overview of conventional bone regeneration treatments, illustrating allografts, autografts, and bone substitutes. The key characteristics of each treatment type are highlighted, and their respective limitations are outlined.

The inherent limitations of conventional bone regeneration methods underscore the need for NFMS. Autografts, while effective, entail donor site morbidity and discomfort, with a finite supply of autologous bone posing constraints, especially for extensive defects or recurring surgeries.^38^ Allografts, despite advances in tissue matching and processing, still carry risks of immune reactions and postsurgical complications, potentially leading to graft failure.^39^ Although readily available and safer in terms of disease transmission, synthetic bone substitutes often lack the intricate biochemical and structural cues inherent in the native ECM. This deficiency impairs their ability to effectively stimulate cellular adhesion, proliferation, and differentiation, which is vital for robust tissue regeneration.^40^ Moreover, both allografts and synthetic substitutes frequently fall short of delivering the requisite mechanical properties necessary to support load-bearing functions in bone. This shortfall can precipitate implant failure, inadequate integration with surrounding tissue, or insufficient stability for optimal healing.^41^ Traditional approaches also offer limited options for tailoring scaffold properties to match the distinct requirements of target tissues or individual patients. Such a “one-size-fits-all” paradigm may inadequately address the diverse needs of patients presenting with varying bone defects or injuries.^42^Figure 2 depicts a conceptual figure illustrating the different types of conventional bone regeneration treatments and their major limitations.

## 3 Designing NFMS

**Figure 3:**
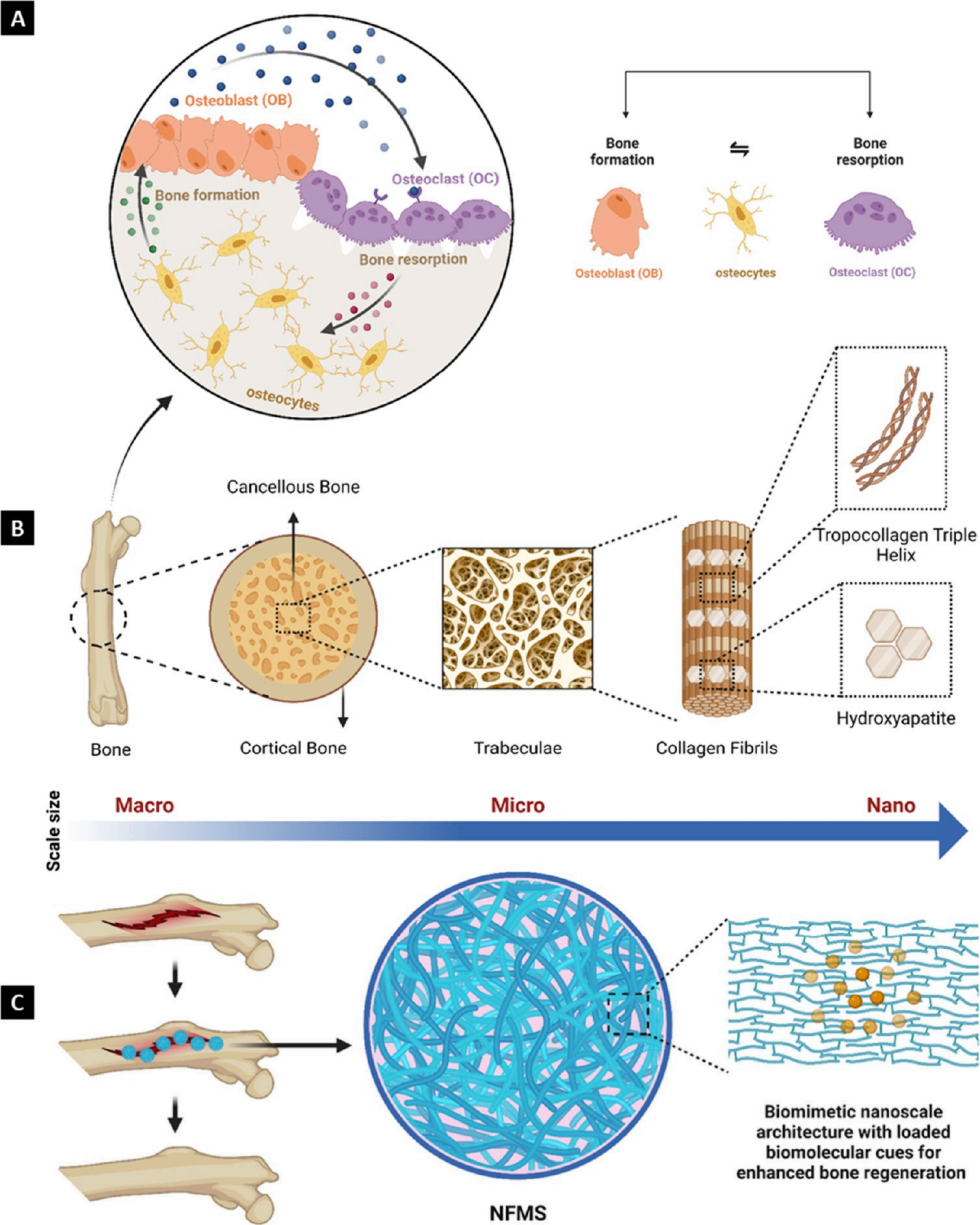
(A) Bone remodeling process. It is a dynamic process orchestrated by osteoclasts, osteoblasts, and osteocytes. Osteoclasts resorb old or damaged bone tissue, while osteoblasts deposit new bone matrix. Osteocytes act as regulators, coordinating the activity of osteoclasts and osteoblasts. In injuries requiring external intervention, such as fractures, bone homeostasis disrupts, necessitating surgical or medical interventions to realign fractured segments, promote bone healing, and restore structural integrity. (B) Main mineral/protein components of bone. Trabeculae are the lattice-like structures found in cancellous or spongy bone, consisting of a network of interconnected rods and plates. They primarily comprise a matrix of tropocollagen triple helices, providing flexibility and hydroxyapatite crystals, imparting strength and rigidity to the bone tissue. Bone regeneration becomes complex due to this variety in composition and mechanical features. (C) Schematic representation of NFMS. NFMS are microscale carriers characterized by nanoscale architectural features. By mimicking the matrix features of bone and delivering bioactive cues, NFMS provides a biomimetic environment conducive to bone regeneration.

The design of NFMS for bone tissue regeneration involves a meticulous selection of active components, optimization of fabrication techniques, and integration of biomimetic cues to replicate the complex microenvironment of native bone tissue. Figure 3 provides an overview of the process of bone remodeling, its components (at micro- and nanoscales), and a schematic diagram of NFMS, highlighting its microscale size with nanofibrous architecture that aids in recreating the bone matrix at the site of injury.

The choice of biomaterial determines the properties and performance of NFMS, influencing factors such as biocompatibility, degradation kinetics, mechanical strength, and bioactivity. Poly(lactic-*co*-glycolic acid) (PLGA) and polycaprolactone (PCL) are commonly used as the matrix material.^43,44^ They provide the structural scaffold necessary for NFMS formation while ensuring compatibility with host tissues.^45,46^ PLGA exhibits a controlled release of encapsulated bioactive molecules and can be tailored to match specific tissue regeneration needs,^47^ while PCL possesses favorable mechanical properties, including low modulus and slow degradation rate, making it suitable for load-bearing applications in bone tissue engineering.^48^ In addition, materials like collagen, gelatin, and hyaluronic acid (HA) have also been explored.^49^ Collagen is a natural protein found abundantly in the extracellular matrix of various tissues, including bone.^50^ NFMS fabricated from collagen offers excellent biocompatibility and bioactivity.^51^ Gelatin is derived from collagen through partial hydrolysis and exhibits similar biocompatibility and bioactivity properties.^52^ Both of these materials facilitate superior cell adhesion, proliferation, and differentiation.^53^ Lastly, HA is a naturally occurring polysaccharide present in the ECM of connective tissues. HA is employed on account of its ability to be functionalized with bioactive molecules or cross-linked with other polymers to enhance mechanical stability and bioactivity.^54^ Other widely used biomaterials include stimuli-responsive derivatives of silk, chitosan, and poloxamers.^55−57^

Incorporating biomolecules into nanofibrous microspheres is crucial for enhancing their bioactivity and therapeutic potential in bone regeneration. Based on their biochemical role and contribution to bone regeneration, these biomolecules can be categorized into the following groups:

- Osteoinductive factors: These are a class of signaling molecules that possess the ability to stimulate the differentiation of undifferentiated cells (such as mesenchymal stem cells; MSCs) into osteoblasts, the bone-forming cells.^58^ They promote bone formation by initiating signaling cascades within precursor cells, leading to their differentiation into osteoblasts and subsequent deposition of bone matrix.^59^ They are typically members of the transforming growth factor-beta (TGF-β) superfamily, with bone morphogenetic proteins (BMPs) being the most well-known and extensively studied example.^60^ BMP-2 and BMP-7 are widely studied for their ability to induce osteogenic differentiation of MSCs and promote bone formation.^61^ They exert their effects by binding to cell surface receptors and activating the Smad intracellular signaling pathway.^62^ Other osteoinductive growth factors include certain isoforms of TGF-β itself and other proteins such as insulin-like growth factor (IGF) and fibroblast growth factor (FGF). TGF-β isoforms, particularly TGF-β1, regulate various aspects of bone homeostasis, including cell proliferation, differentiation, and extracellular matrix synthesis.^63^ It also stimulates the production of osteogenic proteins like osteocalcin and collagen type I.^64^
- Angiogenic factors: These are a class of signaling molecules that stimulate the formation of new blood vessels from pre-existing vasculature, a process known as angiogenesis. They promote endothelial cell proliferation, migration, and tube formation, leading to the formation of functional blood vessels.^65,66^ In bone regeneration, vascular endothelial growth factor (VEGF) promotes vascularization of the scaffold, ensuring adequate oxygen and nutrient supply to regenerating tissues. Various isoforms of VEGF, such as VEGF-A, VEGF-B, and VEGF-C, have been studied for their angiogenic properties.^67^ The use of fibroblast growth factor (FGF), particularly FGF-2 (also known as basic FGF), stimulates angiogenesis and plays roles in cell proliferation, migration, and differentiation. It promotes the recruitment of endothelial progenitor cells and induces the formation of mature blood vessels within the regenerating bone tissue.^68^
- Mineralization-inducing factors: These biomolecules promote the deposition of mineral components, such as calcium and phosphate ions, within tissues. In the context of bone regeneration, they play essential roles in the process of mineralization, which involves the formation of hydroxyapatite crystals within the ECM.^69^ Their use is crucial for enhancing osteogenic differentiation of progenitor cells and facilitating the formation of new bone tissue. Calcium phosphate (CaP) materials, such as hydroxyapatite and tricalcium phosphate (TCP), are commonly used as mineralization-inducing scaffolds due to their similarity to the mineral phase of natural bone.^70^ TCP is more soluble than hydroxyapatite and undergoes gradual resorption, releasing calcium and phosphate ions that promote osteogenesis.^71^ In addition, β-glycerophosphate, which is a precursor for inorganic phosphate, has been shown to enhance mineralization by providing a source of phosphate ions for hydroxyapatite crystal formation.^72^
- Immunomodulatory factors: They modulate the activity of immune cells, such as macrophages, T cells, and regulatory T cells (Tregs), to promote a regenerative immune phenotype.^73^ They enhance the resolution of inflammation, promote the switch from pro-inflammatory (M1) to anti-inflammatory (M2) macrophage phenotypes, and promote the recruitment of immune cells that support tissue repair and regeneration.^74,75^ Interleukin-10 (IL-10) is an anti-inflammatory cytokine that inhibits the production of pro-inflammatory cytokines and promotes tissue repair. It modulates the immune response by suppressing the activation and function of macrophages and T cells.^76^ IL-10 has been shown to enhance bone regeneration by reducing inflammation and promoting a regenerative microenvironment conducive to tissue healing.^74^ Tumor necrosis factor-alpha (TNF-α) inhibitors block the action of TNF-α, a pro-inflammatory cytokine implicated in bone resorption and destruction. In conditions such as rheumatoid arthritis and inflammatory bone diseases, excessive TNF-α production contributes to bone loss and joint destruction.^77^ TNF-α inhibitors, including monoclonal antibodies and soluble receptors, mitigate inflammation and protect against bone damage by suppressing osteoclast activity and promoting bone formation.^78^

Besides biomolecules, the direct incorporation of ECM proteins, such as fibronectin or osteopontin, into NFMS facilitates bone regeneration by providing cell-binding motifs and signaling cues.^79^

In addition to the choice of biomaterial and functional biomolecules to be incorporated, the success of NFMS hinges significantly on the careful selection and optimization of fabrication techniques. Fabrication techniques not only dictate the physical characteristics of NFMS, such as size, morphology, and surface properties, but also influence their mechanical strength, drug loading capacity, and biodegradability.^80,81^ Several techniques have been explored, each offering unique advantages and challenges, necessitating a thorough understanding of their principles. Table 1 provides an overview of these techniques.

**Table 1:**
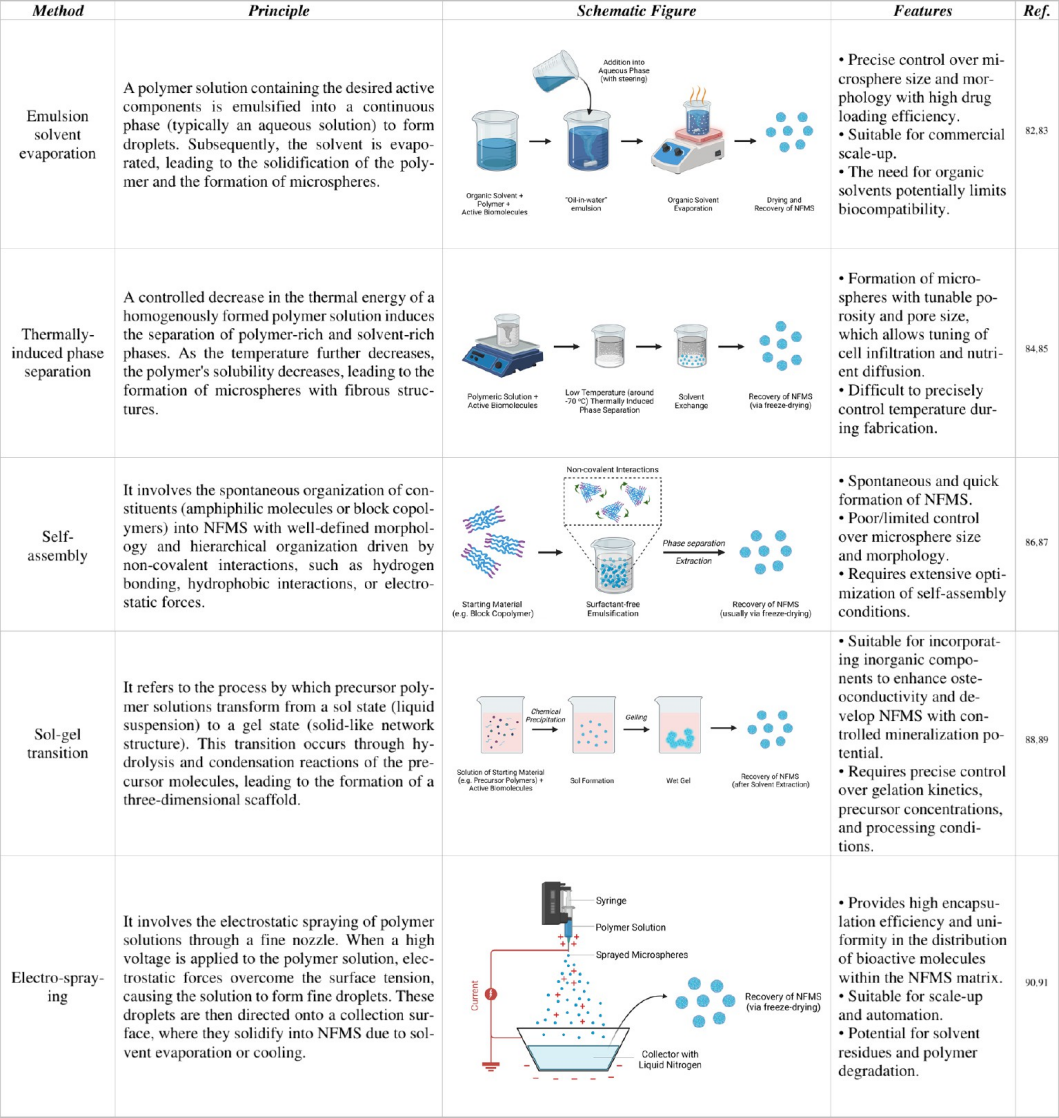
Insights into Different Fabrication Techniques for NFMS^82−91^

Regardless of the technique, proper optimization of formulation parameters is essential to achieve desired NFMS characteristics. One key parameter to consider is polymer concentration, as it directly influences the mechanical properties, porosity, and drug release kinetics of NFMS. Higher polymer concentrations tend to result in microspheres with increased stiffness and mechanical strength, which may be desirable for applications requiring structural support or load-bearing capability.^92^ However, balancing this with considerations such as cell infiltration and nutrient diffusion is essential, as overly stiff microspheres may impede cellular interactions and tissue integration. Conversely, lower polymer concentrations may yield microspheres with reduced mechanical integrity but enhanced porosity, facilitating cell infiltration and nutrient exchange within the scaffold.^93^

Different solvents exhibit varying degrees of solubility for the polymer matrix and active components, leading to differences in solution viscosity and the rate of solvent evaporation during fabrication. This, in turn, impacts the formation of NFMS, with slower solvent evaporation rates generally resulting in larger particle sizes and reduced encapsulation efficiency.^94^ Moreover, solvent volatility and toxicity should be carefully considered to ensure the safety and reproducibility of the fabrication process.^95^ Processing conditions such as temperature, humidity, and stirring speed also play a critical role in NFMS optimization. These parameters affect factors such as polymer chain entanglement, phase separation kinetics, and droplet formation during fabrication, ultimately influencing the resulting microspheres’ morphology, porosity, and drug release profile.^96,97^

**Figure 4:**
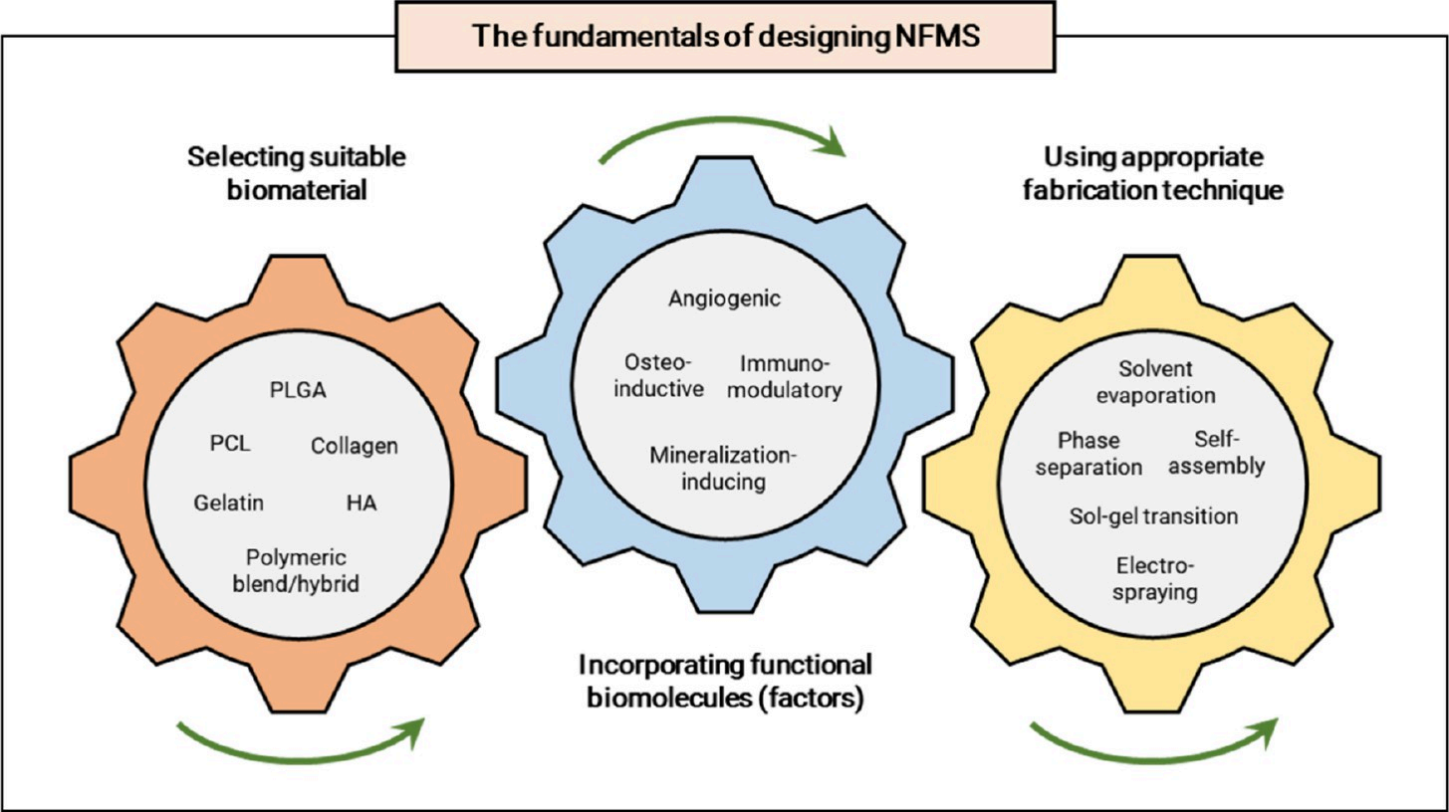
Conceptual schematic representing the interconnected fundamentals of designing NFMS.

Optimization of processing conditions involves systematic experimentation and characterization to identify the optimal combination of parameters that yield NFMS with the desired properties. Figure 4 illustrates the core principles of designing NFMS for bone regeneration applications.

## 4 Key Attributes

Several critical attributes are pivotal for designing effective NFMS for clinical applications. Morphological features, including size, shape, and surface topography, influence cellular interactions and tissue integration. Mechanical properties, such as tensile strength and elasticity, determine the scaffold’s ability to withstand physiological loads and provide structural support. Biocompatibility ensures compatibility with host tissues and minimizes adverse reactions. Moreover, precise characterization of bioactive molecule release kinetics is essential for controlling therapeutic delivery and promoting tissue regeneration. Comprehensive assessment and characterization of these attributes are imperative to tailor NFMS formulations to meet the specific requirements of clinical applications, ensuring optimal performance and safety in regenerative medicine. Here are the key aspects to consider:

### 4.1 Morphological Features

Morphological features refer to the physical characteristics of NFMS. They can vary in size, typically ranging from tens to hundreds of micrometers in diameter. Characterizing the size distribution of NFMS is important as it can impact their behavior in biological systems.^98^ For example, smaller microspheres may exhibit higher cellular uptake rates, while larger microspheres may provide more sustained release of encapsulated bioactive molecules. NFMS can have different shapes, including spherical, ellipsoidal, or irregular.^99^ The shapes of NFMS can influence their packing density, surface area, and interactions with cells and tissues. For instance, spherical NFMS may offer more uniform distribution within a defect site, while ellipsoidal or irregular-shaped NFMS may have enhanced packing efficiency and interlocking capabilities.^100^ Characterizing surface roughness (irregularities or variations in surface texture at micro- and nanoscales) is essential as it can affect cell adhesion and subsequent proliferation/differentiation.^101^ A rougher surface may provide more sites for cell attachment and enhance cellular interactions compared to a smoother surface.^102^ NFMS can be engineered to possess nanoscale topographical features, such as aligned or random nanofibers. Nanotopography mimics the natural architecture of the ECM and guides cell orientation, migration, and behavior.^103,104^ Aligned nanofibers (resembling the orientation of collagen fibers in native tissues) can promote directional cell growth and tissue alignment. In contrast, random nanofibers (resembling the disorganized structure of the ECM) offer an increased surface area for cell adhesion and spreading.^105^ Techniques such as scanning electron microscopy (SEM), atomic force microscopy (AFM), and confocal microscopy are commonly used to visualize and quantify the morphological characteristics of NFMS.

### 4.2 Mechanical Features

The mechanical strength of NFMS is crucial for ensuring their integrity under loading conditions encountered within the body. Tensile strength, which denotes the maximum stress a material can withstand before breaking under tension, is a key parameter evaluated in NFMS. Additionally, the modulus, or stiffness, of NFMS influences their resistance to deformation under applied stress.^106^ Higher modulus values indicate greater stiffness, which may be desirable for providing support in load-bearing applications. Elasticity, the ability of NFMS to return to their original shape after deformation, is also important to prevent permanent damage during dynamic loading. NFMS with high elasticity can undergo deformation without compromising their structural integrity, making them suitable for applications in bone tissue engineering.^107^ In addition to mechanical strength, scaffold stability is paramount for long-term success. The degradation rate of NFMS should match the rate of tissue regeneration to ensure that the scaffold provides support throughout the healing process without impeding tissue integration.^108^ Furthermore, NFMS should exhibit resistance to enzymatic degradation in biological environments to maintain their structural integrity over the desired period. Biomechanical compatibility is another critical aspect to consider when designing NFMS for bone tissue regeneration. NFMS should possess mechanical properties that closely match those of native bone tissue to minimize stress shielding and promote physiological loading transfer.^109^ By mimicking the mechanical properties of trabecular or cortical bone, NFMS can facilitate seamless integration with the surrounding tissue and support natural bone remodeling processes. Evaluating the biomechanical compatibility of NFMS involves assessing their response to dynamic mechanical stimuli and their adaptation to changes in the in vivo environment during tissue regeneration. Through mechanical testing, finite element analysis, and in vivo mechanical evaluations, researchers can thoroughly characterize the mechanical features of NFMS and ensure their suitability for successful bone tissue regeneration applications.^110^

### 4.3 Biocompatibility and Cell Recruitment/Attachment

Assessing cell viability and proliferation provides insights into the compatibility of NFMS with living cells. Cell viability assays, such as MTT or AlamarBlue assays, measure metabolic activity and indicate the extent to which NFMS support cell growth and maintenance.^111^ Additionally, cytotoxicity assays, including lactate dehydrogenase (LDH) release assays and live/dead staining, help determine whether NFMS induces any significant cell death or damage.^112^ Evaluating the inflammatory response elicited by NFMS is essential. Measurements of pro-inflammatory cytokines (e.g., TNF-α, IL-6) and anti-inflammatory cytokines (e.g., IL-10) secretion from immune cells provide insights into the inflammatory potential of NFMS.^113^ Hemocompatibility assessments, such as hemolysis assays, evaluate the interaction of NFMS with blood components, ensuring minimal damage to red blood cells and good hemocompatibility.^114^ Besides biocompatibility, the ability of NFMS to recruit and support the attachment of relevant cell types is crucial for bone tissue regeneration. Cell adhesion assays, which involve seeding cells onto NFMS and assessing their attachment using microscopy techniques, offer valuable insights into the adhesive properties of NFMS.^115^ Adequate cell attachment indicates that NFMS provide a suitable substrate for cell adhesion, an essential prerequisite for tissue integration. Moreover, investigating the involvement of integrin-mediated signaling pathways in cell–NFMS interactions sheds light on the mechanisms underlying cell adhesion, spreading, and signaling.^116^

### 4.4 Release of Biotherapeutics

Encapsulation efficiency, influenced by factors such as the physicochemical properties of the biotherapeutics and the compatibility with the NFMS matrix, is crucial in ensuring optimal therapeutic efficacy. To measure it, NFMS are typically separated from the unencapsulated biotherapeutics using techniques such as centrifugation, filtration, or ultracentrifugation.^117^ The amount of encapsulated biotherapeutics is determined by quantifying the biotherapeutic content within the NFMS. Depending on the nature of the biotherapeutic molecule, various analytical techniques can be employed, such as high-performance liquid chromatography (HPLC) for small molecules or peptides, enzyme-linked immunosorbent assay (ELISA) for proteins or growth factors, fluorescence spectroscopy for fluorescently labeled molecules, and UV–vis spectroscopy for absorbance-based quantification of molecules with chromophores.^118,119^ Prior to quantification, NFMS may need to be dissolved or disrupted to release the encapsulated biotherapeutics, depending on the encapsulation method and the compatibility of NFMS with the analytical technique. Release kinetics of biotherapeutics from NFMS can be precisely modulated to achieve sustained and tailored release profiles.^120^ Characterizing the release kinetics involves monitoring the cumulative release of biotherapeutics over time and elucidating the underlying release mechanisms, such as diffusion-controlled or degradation-controlled release.^121^ The release profile of biotherapeutics from NFMS can be tailored to match specific therapeutic requirements. Strategies such as surface modification, polymer blending, or incorporation of release modifiers (e.g., nanoparticles) can be employed to customize release profiles, enabling precise control over the timing and duration of therapeutic delivery.^122,123^

### 4.5 Osteogenic Properties

When characterizing the osteogenic properties of NFMS, several key aspects must be thoroughly assessed to understand their efficacy in promoting bone formation. First, it is imperative to evaluate their ability to induce osteogenic differentiation of precursor cells (such as MSCs), by examining the expression of osteogenic markers like alkaline phosphatase (ALP), osteocalcin, and RUNX2.^124^ These markers signify the commitment of cells to the osteoblastic lineage and their capacity to produce bone matrix.^125^ Second, the mineralization potential of NFMS should be assessed by examining their ability to facilitate calcium deposition and hydroxyapatite crystal formation. This involves quantifying mineral content and assessing the organization and density of mineral deposits within the NFMS scaffold.^126,127^ Additionally, the expression of genes associated with mineralization, such as those encoding for bone matrix proteins like collagen type I^128^ and bone sialoprotein,^129^ should be analyzed to elucidate the molecular mechanisms underlying mineralization processes. Finally, in vivo studies using animal models of bone defects or injuries can provide valuable insights into the osteogenic potential of NFMS in a physiological environment.^130^ Evaluating the ability of NFMS to promote bone formation, improve bone healing, and integrate with surrounding native tissue can validate their efficacy as bone tissue engineering scaffolds.^131^

### 4.6 Stimuli-Responsiveness

Stimuli-responsiveness encompasses various mechanisms that enable biomaterials to adapt and respond to specific cues in their environment, influencing their behavior and functionality by providing spatiotemporal control over their therapeutic delivery.^132^ NFMS can be engineered to be responsive to different types of stimuli, including pH, temperature, and enzymes. pH-responsive NFMS incorporate polymers or functional groups that undergo conformational changes or dissolution in response to acidic or alkaline conditions, which are prevalent in certain diseased tissues or cellular microenvironments.^133^ Temperature-responsive NFMS utilize thermosensitive polymers such as poly(*N*-isopropylacrylamide) (PNIPAAm), which exhibit a lower “critical solution temperature” close to physiological temperature. These polymers undergo phase transitions or changes in solubility in response to temperature variations, impacting drug release kinetics or scaffold properties.^134^ Enzyme-responsive NFMS contain peptide sequences or chemical moieties susceptible to enzymatic cleavage by specific enzymes like matrix metalloproteinases or proteases present in the tissue microenvironment.^135,136^ Upon enzymatic cleavage, these NFMS can undergo triggered degradation or release of encapsulated bioactive molecules. Characterizing the stimuli-responsiveness of NFMS involves assessing their response to the specific stimulus of interest and quantifying the resulting changes in properties or behavior. For pH-responsive NFMS, characterization may involve evaluating changes in swelling behavior, surface charge, or release kinetics in response to pH variations.^137^ Temperature-responsive NFMS can be characterized by studying changes in polymer conformation, sol–gel transition temperatures, or mechanical properties as a function of temperature.^138^ Enzyme-responsive NFMS are typically characterized by monitoring enzymatic degradation kinetics or release profiles of bioactive molecules in the presence of relevant enzymes.^139,140^

Table 2 provides a comprehensive summary of the critical physical, mechanical, biological, and functional attributes of NFMS, highlighting their significance in the context of bone regeneration.

**Table 2:** Insights into Key Attributes of NFMS for Clinical Applications in Bone Tissue Regeneration

Attribute	Subcategory	Details/Values	Measurement Technique	Implications/Notes
Morphological features	Size	50–500 μm in diameter	DLS, SEM	Size affects cellular behavior and bioactive molecule release rates. Optimal size ranges for different applications have been identified based on target tissue and cell type.
	Shape	Spherical, ellipsoidal, irregular	SEM, 3D imaging techniques	Spherical shapes offer consistent bioactivity and ease of injection; ellipsoidal shapes maximize packing density and mechanical interlock within defect sites.
	Surface topography	Nanofiber alignment (aligned or random)	SEM, AFM	Aligned nanofibers promote directional tissue growth resembling natural ECM orientation, enhancing tissue structural organization and function.
Mechanical features	Tensile strength	1–10 MPa	Universal testing machines (tensile testing)	Essential for load-bearing applications; the tensile strength should match or exceed that of surrounding tissue to prevent mechanical failure.
	Elasticity	High elasticity, capable of 50–100% strain	Tensile testing, rheometry	Critical for maintaining structural integrity in dynamic environments, especially in orthopedic applications where deformation is frequent.
	Modulus (stiffness)	0.1–10 GPa	Nanoindentation, mechanical testing	Stiffness tailored to mimic specific bone types (e.g., trabecular vs cortical), optimizing integration and minimizing stress shielding effects.
Biocompatibility	Cell viability	90–95% viability in cell cultures	MTT assay, AlamarBlue assay	High viability rates are indicative of excellent biocompatibility, essential for clinical approval and successful integration.
	Cytotoxicity	LD_50_ values, standardized cytotoxicity ratings	ISO standard cytotoxicity tests, LDH assay	Quantitative assessment of cytotoxicity provides a benchmark for safety, essential for regulatory approval and clinical use.
	Inflammatory response	Low TNF-α, IL-6; high IL-10 secretion	ELISA, multiplex cytokine Assay	Optimized NFMS formulations exhibit reduced pro-inflammatory responses and enhanced anti-inflammatory properties, suitable for sensitive applications like implantation.
Release of biotherapeutics	Encapsulation efficiency	Typically 70–90% efficiency	Spectrophotometry	High encapsulation efficiency is crucial for dose control and sustained release, significantly impacting therapeutic outcomes.
	Release kinetics	Controlled release over 1–4 weeks	In vitro release studies, HPLC	Release kinetics can be engineered to match the therapeutic window of the bioactive agent, crucial for chronic conditions.
Osteogenic properties	Biomarkers	Increased expression of ALP, osteocalcin, and RUNX2	Immunohistochemistry, qPCR for gene expression	Marker expression correlates with osteogenic activity, providing measurable end points for evaluating NFMS efficacy in promoting bone regeneration.
	Mineralization	Enhanced calcium and phosphate deposition	Alizarin Red staining, Von Kossa staining	Directly linked to the structural and functional integration of the bone repair process, critical for long-term success in bone healing.
Stimuli- responsiveness	pH	Trigger release at pH 6.5–7.4	pH-sensitive dye labeling, real-time pH monitoring	Ideal for targeted release in slightly acidic environments of inflamed or tumorous tissues, enhancing local therapeutic impact.
	Temperature	Activation at 37–42 °C	Differential scanning calorimetry, thermogravimetric analysis	Utilized for on-demand drug release triggered by slight increases in local body temperature, common in inflamed or regenerating tissues.
	Enzyme	Enzyme specific cleavage	Gel zymography, in vitro enzymatic degradation studies	Enables localized release in response to enzymatic activity typical of remodeling tissues, providing highly targeted therapy.

## 5 Applications in Bone Regeneration: Preclinical Case Studies

This section explores contemporary preclinical studies utilizing NFMSs for advancing bone regeneration.

**Figure 5:**
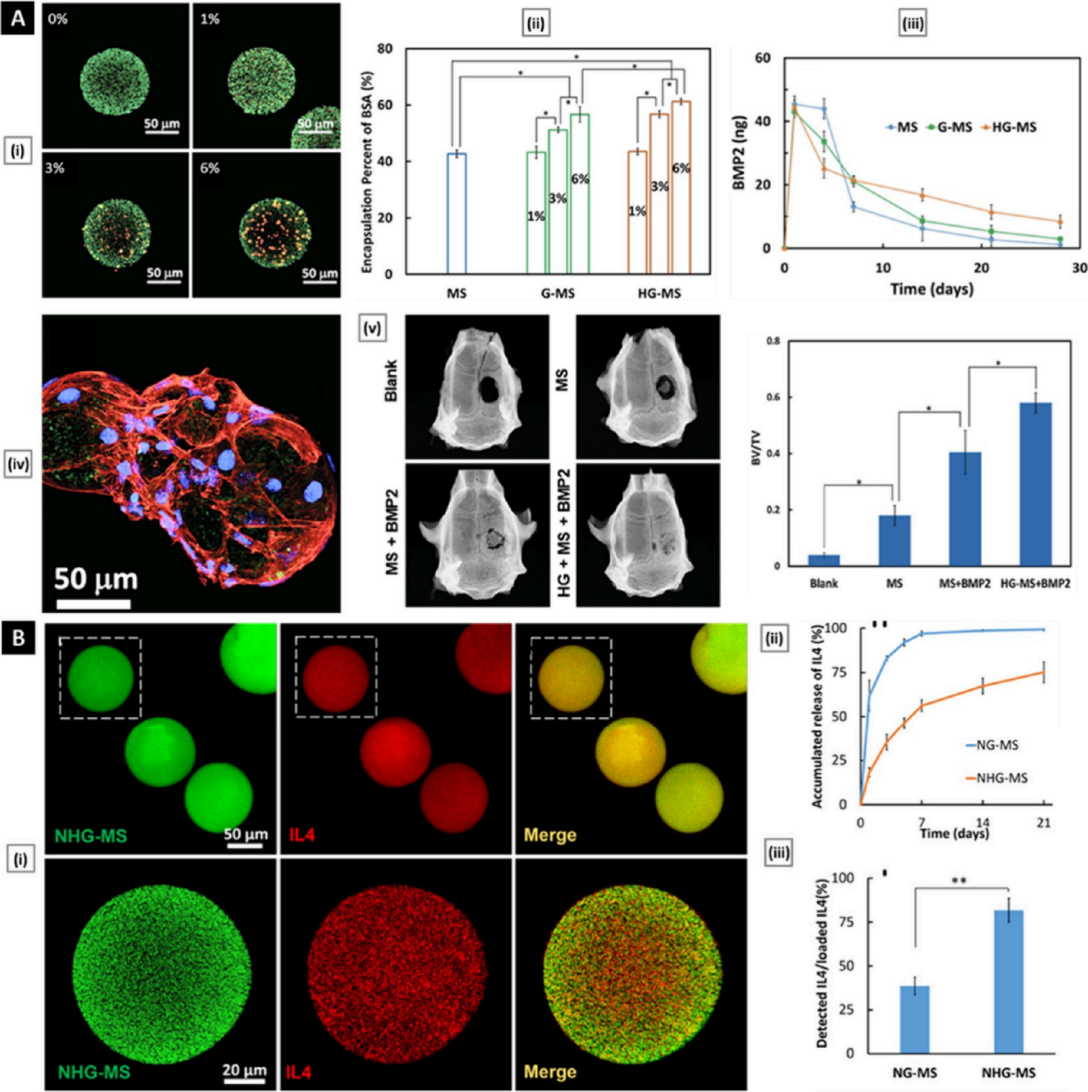
(A) Hierarchical NFMS with controlled growth factor delivery for bone regeneration. Panel A(i) shows confocal images of HG-MS fabricated with different ratios of HG/PLLA. Panel A(ii) shows the encapsulation percent of BSA in microspheres fabricated with different ratios of HG/PLLA [*p < 0.05]. Panel A(iii) shows the release profile of BMP2 (500 ng/mg MS) from MS, G-MS, and HG-MS. Panel A(iv) shows a confocal image of BMSCs adhering to HG-MS. The action of the BMSCs was labeled red, and the nuclei of the BMSCs were labeled blue. Panel A(v) shows X-ray images and the corresponding BV/TV ratio of the calvarial bony defects 6 weeks after implantation [*p < 0.05]. Reproduced with permission from ref (141). Copyright 2015 Wiley-VCH. (B) Immunomodulatory ECM-like microspheres for accelerated bone regeneration in diabetes mellitus. Panel B(i) shows stacked confocal images of IL4-loaded NHG-MS and cross-sectional images at a higher magnification. IL4 (red) was evenly distributed in the NHG-MS (green). Panel B(ii) shows the release profiles of IL4 from NHG-MS and NG-MS. Panel B(iii) shows the total amount of IL4 (released and unreleased) from the NHG-MS and NG-MS detected via an ELISA [**p < 0.01]. Reproduced with permission from ref (143). Copyright 2017 American Chemical Society.

Ma et al.^141^ pioneered a hierarchical scaffolding system for bone tissue regeneration, featuring heparin-conjugated gelatin (HG) to bind bone morphogenetic protein 2 (BMP2). Utilizing a water-in-oil-in-oil (W/O/O) double emulsion technique, coupled with chemical cross-linking and thermally induced phase separation, BMP2-binding HG nanospheres are encapsulated within NFMS, forming HG-MS. The biomimetic system, characterized by self-assembled synthetic nanofibers (366 ± 146 nm diameter), mirrors natural collagen fibers, offering high porosity (94.24% ± 0.52%), low density, and superior surface area for optimal cell adhesion and tissue in-growth. This unique system combines heparin-binding and nanosphere encapsulation, leading to a sustained release profile. In vitro studies using bovine serum albumin (BSA) demonstrate a reduced initial burst release in HG-MS compared to counterparts lacking nanospheres. Quantitative analysis reveals sustained release over weeks, with 17% of BMP2 released during the second to fourth weeks, demonstrating controlled delivery efficacy. Bioactivity assays with rat bone marrow stem cells (BMSCs) confirm BMSC adhesion to HG-MS and highlight unaffected proliferation rates despite BMP2 loading, emphasizing a selective impact on differentiation and mineralization. Real-time polymerase chain reaction analysis indicates increased expression of osteogenic markers on BMP2-loaded HG-MS, affirming growth factor bioactivity. Translational potential is emphasized through in vivo assessments using a rat calvarial bone defect model. X-ray and microcomputed tomography (μ-CT) images depict enhanced bone formation in BMP2-loaded HG-MS-treated sites, surpassing control group outcomes. Superior bone volume to total volume (BV/TV) ratios were observed in BMP2-loaded HG-MS specimens (Figure 5A). Histological analyses, including H&E and Masson’s trichrome staining, consistently show superior outcomes with BMP2-loaded HG-MS. Immunohistochemistry supports these findings, revealing heightened expression of dentin matrix acidic phosphoprotein 1 (DMP1) and collagen type 1 (Col1) in the BMP2-loaded HG-MS group.

In a similar study focusing on BMP-2, Wang et al.^142^ investigated the use of NFMS to induce odontogenic differentiation in human stem cells of the apical papilla (SCAP). Dental lesions and loss from caries, periodontal diseases, and trauma necessitate regenerative therapies due to limitations in traditional restorative materials. Addressing this challenge, the authors meticulously integrated scaffold design with the controlled release of BMP-2 to orchestrate a highly effective dental tissue regeneration strategy. NFMS exhibited a nanofibrous architecture resembling collagen fibers, boasting an average diameter of approximately 160 nm. SCAP, strategically selected for their anatomical positioning and demonstrated proficiency in dentin-like tissue formation, exhibited a noteworthy response to BMP-2 treatment. In vitro experiments unequivocally illustrated heightened odontogenic differentiation, manifested by increased alkaline phosphatase (ALP) activity, elevated calcium content, and up-regulated expression of odontogenic genes (Col I, BSP, OCN, and DSPP). Advancing into a 3D spinner flask culture system, the study showcased the sustained efficacy of BMP-2 on NFMS, underscoring its potential for clinical applications. NFMS, based on PLGA, facilitated the controlled release of BMP-2, ensuring sustained and dose-dependent effects. In vivo evaluations further validated the success of this approach, with SCAP and NFMS subcutaneously implanted, exhibiting dentin-like tissue formation, mineralization, and robust DSPP protein expression. This investigation not only unveils the intricate interplay between scaffold architecture, growth factor delivery, and SCAP response but also establishes a solid foundation for future endeavors in refining injectable biomaterials.

In an innovative research effort, the critical challenge of bone regeneration in diabetes mellitus (DM) was addressed by Hu et al.^143^ Through the development of immunomodulatory ECM-inspired gelatin-based NFMS (termed NHG-MS), the research authors aimed to modulate the immune microenvironment and expedite bone healing under diabetic conditions. The fabrication process involved an emulsification and phase separation technique, yielding a nanofibrous architecture. The gelatin used in the study was modified with heparin to facilitate the controlled incorporation and release of Interleukin 4 (IL4), a crucial anti-inflammatory cytokine essential for macrophage polarization. The prepared NHG-MS showed high porosity (95.0 ± 0.4%) and low apparent density (0.075 g/cm^3^), both crucial for optimal cellular responses. It demonstrated enhanced IL4 loading efficiency compared to non-heparin-conjugated counterparts (Figure 5B). Utilizing ELISA, the platform demonstrated a sustained release profile of IL4 over a period of 3 weeks. This prolonged delivery, attributed to the protective role of heparin in preventing IL4 degradation, highlights the controlled nature of cytokine release. In vitro bioactivity assays further confirmed the ability of IL4 released from NHG-MS to effectively repolarize pro-inflammatory M1 macrophages into an anti-inflammatory M2 phenotype, substantiating its potent immunomodulatory capabilities. Validation of the platform extended to an in vivo rat mandibular periodontal fenestration model simulating DM condition. Here, IL4-loaded NHG-MS significantly reduced inflammatory cytokines, polarized macrophages, and enhanced osteogenesis. Histological and microcomputed tomography (μ-CT) analyses provided additional confirmation of the microspheres’ potential to foster bone regeneration under diabetic conditions. This study enhances the understanding of the intricate interplay between inflammation and bone healing in DM, introducing a versatile immunomodulatory biomaterial with broad implications for tissue engineering and regenerative medicine.

**Figure 6:**
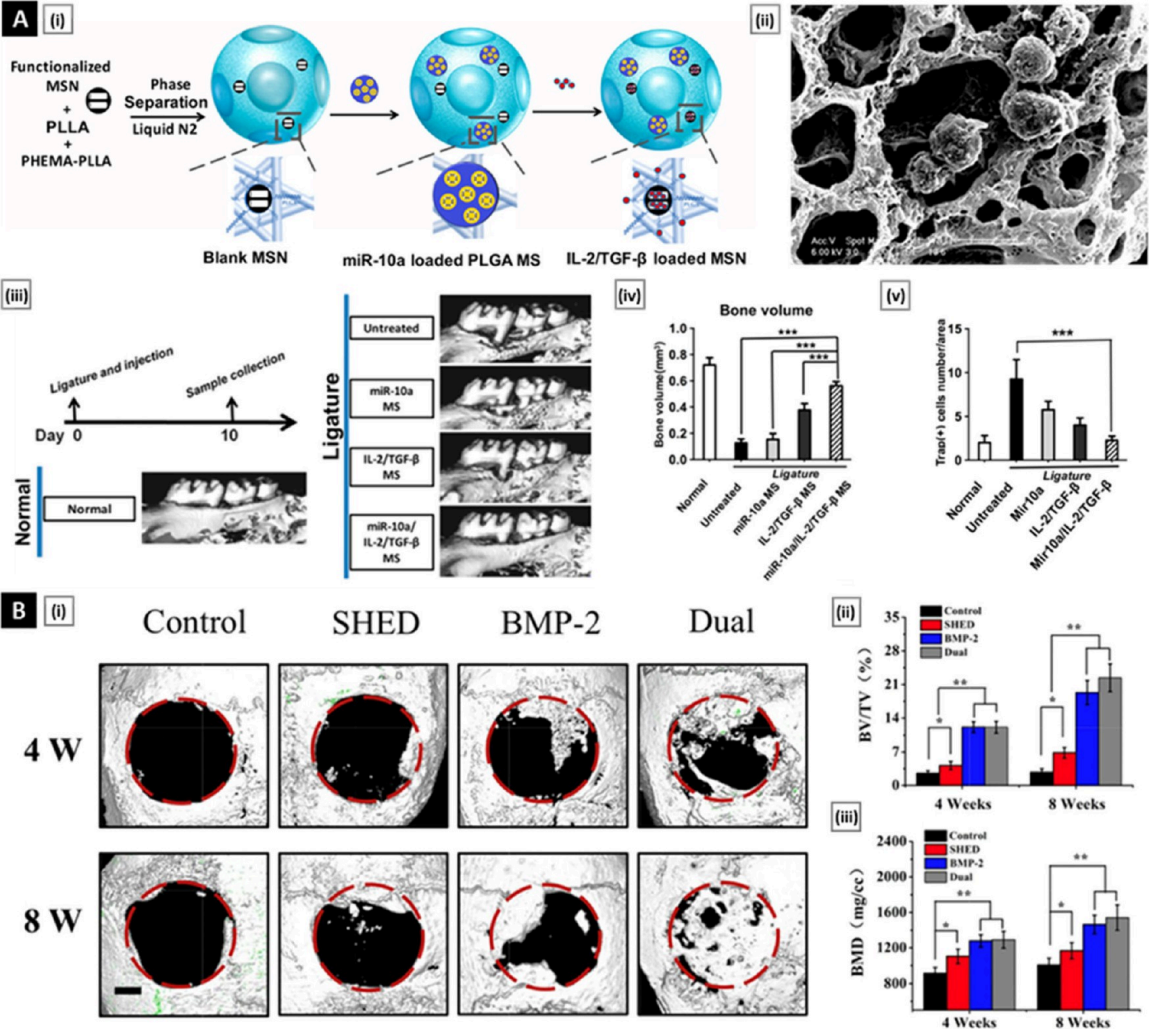
(A) miRNA and growth factors-loaded spongy NFMS to rescue periodontal bone loss. Panel A(i) shows an illustrative flowchart of fabricating multifunctionalized PLLA NFMS with MSN to incorporate growth factors and PLGA MS to incorporate microRNA/HP polyplexes. Panel A(ii) shows T cells in multifunctionalized NFMS observed SEM. Panels A(iii) and A(iv) show μ-CT results of bone loss between the first and second molars in the periodontitis model and corresponding changes in the bone volume for various treatment groups. Panel A(v) shows the quantification of TRAP-positive cells in the maxillae of mouse periodontal disease mode [in this study, differences were considered statistically significant if *p* < 0.05]. Reproduced with permission from ref (144). Copyright 2018 American Chemical Society. (B) Synergistic effect of stem cells from human exfoliated deciduous teeth and rhBMP-2 delivered by injectable NFMS. Panel B(i) shows reconstructive 3D μ-CT photographs of repaired cranial bone defects in all groups at 4- and 8-weeks postoperation (scale bar, 1 mm) in nude mice. Panels B(ii) and B(iii) show quantitative analysis of BV/TV and BMD values for four groups [*p < 0.05, **p < 0.01]. Reproduced with permission from ref (145). Copyright 2019 Elsevier.

In the investigation conducted by Liu et al.,^144^ a novel NFMS-based scaffold design was employed, coupled with precise delivery of biologic molecules, shedding light on fundamental mechanisms governing regulatory T cell (Treg) differentiation. The therapeutic efficacy of this innovative approach in mitigating periodontal bone loss was convincingly demonstrated. The multifaceted NFMS, tailored for alveolar bone regeneration, effectively integrated mesoporous silica nanoparticles (MSN) for accelerated release of interleukin-2 (IL-2) and transforming growth factor-beta (TGF-β), along with PLGA microspheres for sustained release of microRNA-10a (miR-10a). Demonstrating excellent biocompatibility and controlled release profiles, NFMS showcased their potential as a carrier for loaded biomolecules. In vitro investigations corroborated the biocompatibility and Treg-inducing capacity of the NFMS. The miR-10a/IL-2/TGF-β-releasing NFMS effectively prompted Treg differentiation, evident from the heightened expression of Foxp3. Functional assays underscored the immunomodulatory prowess of induced Tregs, as they successfully restrained the proliferation of naive T cells. Translating these findings into an in vivo context, the study utilized a murine model of periodontal disease induced by ligature. Notably, the administration of miR-10a/IL-2/TGF-β-releasing NFMS surpassed control interventions in rescuing periodontitis-associated alveolar bone loss. This success highlighted a synergistic effect between miR-10a and IL-2/TGF-β. Gene expression analysis and flow cytometry data provided additional support, demonstrating the modulation of inflammatory cytokines and the induction of CD4 + Foxp3 + Tregs. The elevated Treg/Th17 cell ratio indicated the establishment of a favorable immune regulatory microenvironment. Histological assessments employing H&E, osteocalcin (OCN), and tartrate-resistant acid phosphatase (TRAP) staining validated the therapeutic potential of the NFMS. The miR-10a/IL-2/TGF-β-releasing NFMS effectively rescued alveolar bone loss, as evidenced by restored alveolar bone height, heightened OCN-positive staining, and reduced numbers of TRAP-positive cells (Figure 6A). Collectively, these outcomes underscore the promising role of the developed NFMS as a versatile therapeutic platform for periodontal bone regeneration, particularly in the context of diabetic conditions.

In a different investigation conducted by Fang et al.,^145^ the focus was directed toward optimizing strategies for vascularized bone regeneration through the combined application of stem cells derived from human exfoliated deciduous teeth (SHED) and recombinant human bone morphogenetic protein-2 (rhBMP-2). This was accomplished by utilizing injectable NFMS with distinctive surface modifications. Notably, surface engineering involved the application of a mussel-inspired polydopamine (PDA) coating on PLLA NFMS, resulting in heightened hydrophilicity and improved biocompatibility. The resulting PDA-NFMs exhibited a uniform distribution of nanoscale PDA particles on the fiber surface, influencing enhanced cellular adhesion and proliferation. An innovative modification included the integration of heparin–dopamine (Hep-Dopa) conjugation into NF-Ms, facilitating the controlled release of rhBMP-2. Surface characterization using SEM and EDS confirmed the success of these modifications. Water contact angle measurements demonstrated increased hydrophilicity in PDA-NFMs. The distribution of RBITC-labeled rhBMP-2 evidenced the effective immobilization of rhBMP-2 by Hep-Dopa NF-Ms. Controlled release profiles demonstrated reduced initial burst release and sustained release over 28 days, indicating the potential for sustained and controlled rhBMP-2 delivery. The investigation explored the growth and proliferation of SHED on PDA-NF-Ms, revealing enhanced cellular adhesion, spreading, and ECM secretion. Viability assessments, conducted through live/dead staining and cytoskeleton staining, affirmed the favorable environment provided by PDA-NF-Ms for SHED. Osteogenesis effects of rhBMP-2 released from Hep-Dopa NF-Ms were examined using alkaline phosphatase (ALP) activity, Alizarin Red S (ARS) staining, and gene expression analyses on bone marrow-derived mesenchymal stem cells (BMSCs). Results indicated sustained release of rhBMP-2, promoting ALP production, mineral deposition, and upregulation of osteogenic genes (ALP, Runx2, Col1). Ectopic subcutaneous implantation revealed the long-term survival and pro-angiogenic behavior of SHED. Orthotopic bone regeneration studies, utilizing μ-CT and histological analyses, highlighted the potent osteoinductive effects of rhBMP-2/Hep-Dopa NF-Ms, with enhanced vascularized new bone formation (Figure 6B).

**Figure 7:**
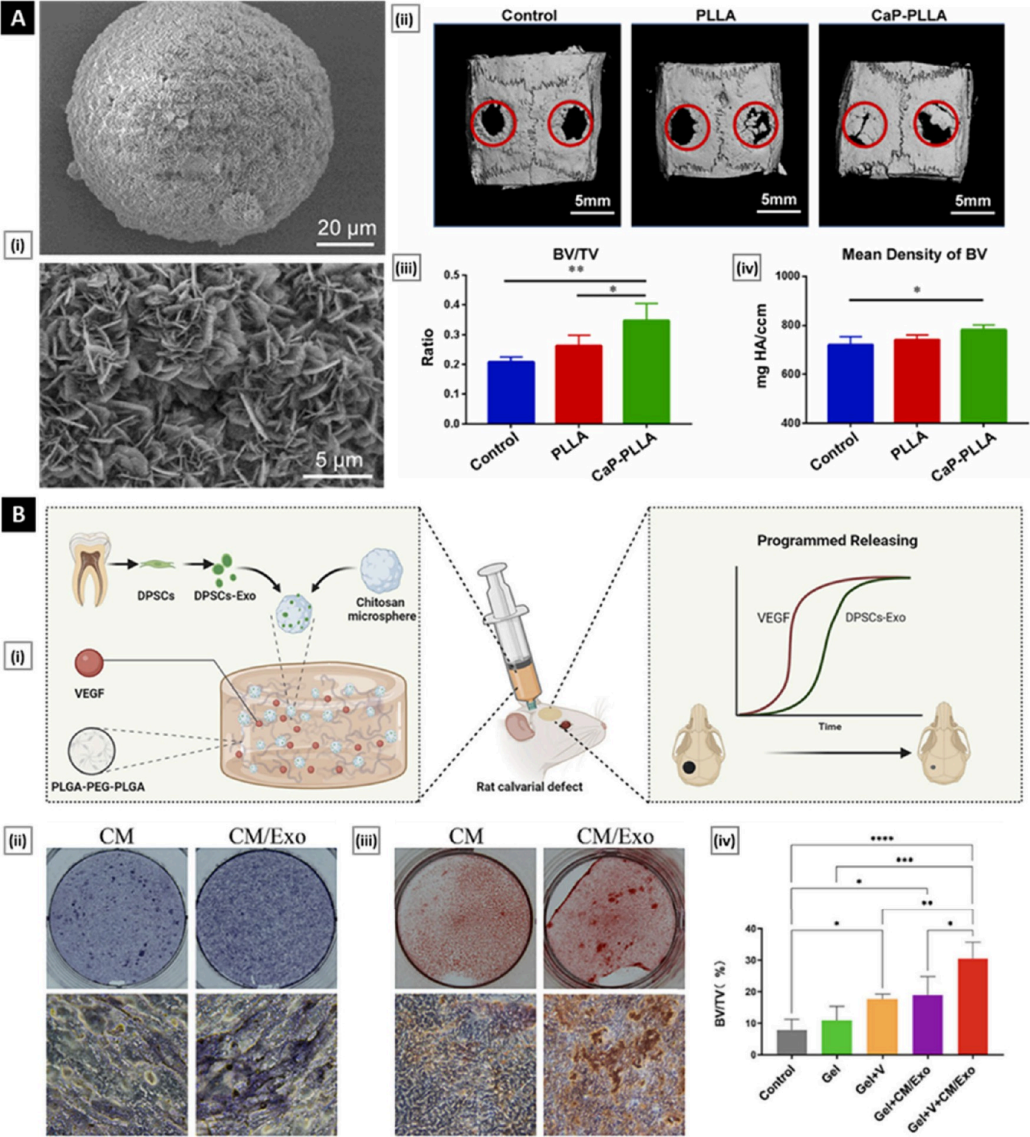
(A) Biomimetic mineralization of PLLA NFMS for bone regeneration. Panel A(i) shows an SEM image of PLLA NFMS treated with STMP and immersed in 2.5 × SBF for 6 days. Panel A(ii) shows 3D reconstructed μ-CT images of rat cranial bone and magnified images of bone defects at 6 weeks following surgery. The red circles indicate the created critical-sized 5 mm defects. Panels A(iii) and A(iv) show bone volume fraction (BV/TV) and bone density analysis at 6 weeks postsurgery [*p < 0.05, **p < 0.01]. Reproduced with permission from ref (146). Copyright 2022 Elsevier. (B) Programmed release of VEGF and exosome from injectable chitosan NFMS-based hydrogel. Panel B(i) shows a schematic of an injectable microsphere-based hydrogel hybrid system capable of the programmed release of VEGF and DPSCs-derived exosomes for enhanced bone regeneration. Panel B(ii) shows ALP staining and ALP activity quantification assay in preosteoblasts at day 14. Subfigures (iii) show Alizarin red staining and quantification of the staining after 21 days of differentiation. Panel B(iv) shows the quantitative analysis of BV/TV value for different treatment groups [***p < 0.001, **p < 0.01, and *p < 0.05]. Reproduced with permission from and (147). Copyright 2023 Elsevier.

In a recent study, Li et al.^146^ explored a novel approach to enhance the osteogenic potential of PLLA NFMS for bone regeneration. Commencing with a comprehensive characterization of PLLA microspheres, the authors delineated their uniform, spherical structure comprising nanofibers measuring approximately 80 μm and 100 nm, respectively. These microspheres served as templates for subsequent surface mineralization. Submerging the microspheres in 2.5 × simulated body fluid (SBF) for 3 to 6 days induced noteworthy morphological transformations. Microspheres treated with sodium trimetaphosphate (STMP) modification exhibited abundant flower-like crystals resembling bone-like apatite. Elemental composition analysis via EDS validated the presence of calcium and phosphorus on the surface, affirming successful biomimetic mineralization. Both nanofibrous PLLA microspheres, with and without surface mineralization, demonstrated significantly heightened cell proliferation compared to dense PLLA microspheres, emphasizing the favorable impact of nanofibrous structures on cellular activity. Additionally, quantitative assessment using the CCK-8 assay revealed sustained disparities in cell proliferation between the modified and unmodified microspheres over a 5 day period. Gene expression profiles were scrutinized through quantitative qPCR to evaluate osteogenic differentiation potential. Surface-mineralized PLLA and nanofibrous PLLA microspheres exhibited markedly elevated expression levels of osteoblast-specific markers, such as osteopontin (OPN), runt-related transcription factor 2 (RUNX-2), and osteocalcin (OCN), in contrast to dense-smooth PLLA microspheres. This signified the active promotion of BMSCs’ commitment to osteogenic lineages by the modified microspheres. μ-CT scanning was employed for a quantitative appraisal of new bone formation in rat calvarial defects. The calcium phosphate–PLLA (CaP-PLLA) group manifested significantly greater new bone formation within 6 weeks, evidenced by higher bone volume fraction (BV/TV) and bone mineral density (BMD) compared to control groups (Figure 7A). Histological analysis further substantiated these results, illustrating well-vascularized new bone structures in the CaP-PLLA group as opposed to fibrous tissue in the control groups.

Han et al.’s^147^ recent investigation introduced an injectable thermosensitive hydrogel system featuring chitosan NFMS. This system was designed for the controlled release of vascular endothelial growth factor (VEGF) and exosomes derived from dental pulp stem cells (DPSCs-Exo). The nanofibrous microspheres were meticulously synthesized using a NaOH/urea aqueous system, resulting in a porous structure ideal for encapsulating and releasing DPSCs-Exo. Previous research has demonstrated the commendable biocompatibility of these microspheres through MTT assays and live/dead cell staining. SEM analysis provided detailed images depicting the microspheres’ spherical morphology, homogeneous surface, and crucially, the nanofibrous structure that enhances drug-loading capacity. The rapid initial water absorption (556.83% within 12 h) and subsequent enzymatic degradation (52.29–73.68% over 21 days) underscore the microspheres’ suitability for sustained release of osteogenic factors. This aligns with the overarching goal of achieving a programmed release of VEGF and DPSCs-Exo, mirroring the dynamic processes of angiogenesis and osteogenesis. Release kinetics were quantified through ELISA, revealing a rapid burst release of VEGF (∼70%) within the initial 7 days, followed by a gradual release over 3 weeks, reaching approximately 96% by day 30. The DPSCs-Exo, encapsulated within the chitosan microspheres, exhibited a more restrained release, with only 9.9% released in the first 7 days and approximately 87.4% over the subsequent 3 weeks. This dual-drug programmed release pattern ensures a temporally orchestrated sequence, crucial for effective bone regeneration. In vitro experiments showcased the hydrogel’s prowess in promoting angiogenesis, as evidenced by tube formation assays with human umbilical vascular endothelial cells, and fostering osteogenic differentiation, demonstrated by ALP and Alizarin red staining. qRT-PCR results highlighted increased expression of angiogenesis-related genes, including Pdgf-β and Ang-1, in Gel + V and Gel + V + CM/Exo groups (Figure 7B). In vivo experiments employing a rat calvarial defect model underscored the outstanding bone regeneration in the Gel + V + CM/Exo group, supported by μ-CT quantification, histological analysis, and immunofluorescence staining for CD31, emphasizing enhanced vascularization.

## 6 Translational Considerations

### 6.1 Market Potential

The global population is aging, leading to a rise in conditions like osteoporosis and osteoarthritis. This demographic shift results in increased demand for treatments promoting bone regeneration, as older individuals often experience bone loss and reduced density.^148^ Musculoskeletal disorders, such as fractures, non-unions, and bone defects from trauma or diseases, further necessitate effective bone regeneration therapies.^149^ Ongoing research in regenerative medicine, tissue engineering, and biomaterials has spurred innovative developments in this field.^150^

Recent data indicate that the global bone regeneration material market reached an estimated value of US$ 2.61 billion in 2023, with an anticipated compound annual growth rate of 4.6% from 2023 to 2030. Guided bone regeneration currently dominates this market, encompassing bone graft substitutes (allograft, xenograft, or synthetic) and barrier membranes (e.g., expanded polytetrafluoroethylene or high density polytetrafluoroethylene).^151^ As discussed previously, traditional bone grafts may fall short in replicating native bone architecture, potentially leading to suboptimal tissue integration. This gap highlights the potential of NFMS in bone tissue regeneration. NFMS offers distinct advantages, including reduced recovery time compared to traditional treatments. Their biomimetic properties enhance cellular interactions, expediting tissue regeneration. Moreover, NFMS demonstrate the potential for fewer postprocedure complications due to their biocompatible design, minimizing infection or immune rejection risks.^152^ The controlled release capabilities of NFMS further enhance their cost-effectiveness, streamlining the treatment process and delivering substantial cost savings for both healthcare systems and patients.^153^

To strategically commercialize NFMS, a comprehensive approach is essential. Licensing agreements can facilitate market entry by sharing fabrication techniques and unique biomaterial formulations. Strategic partnerships should prioritize joint research and development efforts, incorporating specialized expertise in NFMS manufacturing. Establishing in-house manufacturing capabilities requires detailed plans for scaling up production, ensuring quality control, and optimizing cost efficiency.^154^ Technical collaboration with medical device companies involves aligning NFMS specifications with industry standards, ensuring seamless integration into existing healthcare systems. These considerations are crucial for executing effective commercialization strategies within the intricate healthcare market.^155^

### 6.2 Manufacturing and Scale-up

The optimization of manufacturing parameters is a pivotal undertaking in the commercial production of NFMS. Central to this process is the careful consideration and adjustment of various factors. Researchers explore different formulations of polymers, solvents, and bioactive agents to achieve the desired physical and chemical properties. Additionally, optimization extends to temperature and processing time, crucial variables that impact the formation of well-defined nanofibrous structures.^156^ In cases where electrospraying is employed, parameters like voltage, flow rate, and needle-to-collector distance require careful optimization to ensure uniform fiber deposition and prevent irregularities.^157^ The selection and optimization of cross-linking methods, where applicable, further enhance the stability and biocompatibility of NFMS. This optimization is an iterative journey, marked by systematic adjustments based on initial results, ultimately fine-tuning the fabrication process to achieve an efficient and biomimetic platform tailored to the specific requirements of bone tissue regeneration applications.^158,159^

The implementation of quality control measures is a critical component to ensure the reliability, safety, and effectiveness of the final product. The first step involves a detailed characterization of the morphology, encompassing an assessment of size, shape, and surface characteristics.^160^ Techniques such as SEM and AFM provide intricate insights into the physical structure of the microspheres. Size distribution analysis is paramount to guaranteeing uniformity, employing methods like dynamic light scattering or laser diffraction.^161^ Chemical composition analysis, conducted through in-line Fourier-transform infrared spectroscopy, can help ensure the identity and purity of the materials used.^162^ As NFMS is intended for biomedical applications, biocompatibility assessments are undertaken to evaluate interactions with biological systems, ensuring the absence of adverse reactions. Sterility assurance measures, including sterility testing, need to be implemented to confirm the absence of microbial contamination.^163^ Continuous monitoring ensures batch-to-batch consistency, employing statistical analysis and quality control checks to detect and rectify variations in the production process.^164^

The selection and procurement of raw materials significantly impact the properties and performance of NFMS. High quality materials are essential to ensure biocompatibility, mechanical integrity, and therapeutic efficacy. The sourcing process involves evaluating suppliers, assessing material purity, and establishing reliable supply chains to guarantee consistency in the manufacturing process.^165^ Simultaneously, cost considerations play a pivotal role in determining the economic feasibility of NFMS production. Strategies to optimize costs may involve exploring alternative materials with comparable properties, maximizing the efficient use of raw materials, and adopting streamlined manufacturing processes.^166^ Balancing the need for quality materials with cost-effectiveness is essential to develop a sustainable production model that not only meets regulatory standards but also ensures the affordability and accessibility of NFMS-based solutions for bone tissue regeneration.

### 6.3 Regulatory Challenges

Navigating the regulatory landscape for NFMS can present several challenges that may vary based on regional regulatory frameworks. NFMS, with its unique properties and applications, may not neatly align with existing regulatory categories, introducing intricacies in the classification process.^167^ To address this challenge, early engagement with regulatory agencies is paramount, allowing developers to seek guidance and discuss the distinctive features of NFMS for accurate categorization. Thorough characterization studies become instrumental in presenting a comprehensive understanding of NFMS’s technological and functional aspects, aiding regulatory authorities in their classification decision.^168^ Additionally, conducting risk assessments, aligning with established standards, collaborating with regulatory experts, and referencing precedent cases contribute to a more informed classification process. Developers may also advocate for a new regulatory category if NFMS represents a groundbreaking technology that does not fit within existing frameworks.^169^

Designing clinical trials that meet regulatory standards and adequately assess NFMS’s therapeutic effectiveness requires careful consideration. Challenges may arise in determining appropriate end points that capture the sustained benefits of NFMS over an extended period. Additionally, ensuring patient inclusion criteria that align with the intended use and characteristics of NFMS presents a complex task.^170^ To address these challenges, a comprehensive clinical trial design should incorporate well-defined end points that measure both short-term and long-term outcomes. Consideration of patient selection criteria should involve collaboration with clinicians to identify specific patient populations that stand to benefit most from NFMS.^171^ Demonstrating the long-term efficacy and safety of NFMS necessitates prolonged follow-up periods, introducing logistical challenges in maintaining patient participation and data collection over extended durations. Potential solutions include the implementation of patient retention strategies, such as incentives and continuous communication, to encourage participant commitment.^172^ Additionally, leveraging real-world evidence and postmarket surveillance can complement clinical trial data, providing valuable insights into the long-term performance of NFMS in diverse patient populations.^173^

Research on NFMS is still in the early stages, and there are currently no NFMS-based platforms undergoing clinical trials for regenerative therapy. In contrast, conventional microspheres have been extensively developed and are now being integrated into clinical settings, primarily for targeted therapeutic applications such as oncology. This includes the use of radioembolization microspheres to treat hepatocellular carcinoma and biodegradable microspheres to deliver drugs to malignant gliomas.^174,175^ These applications highlight the ability of microspheres to concentrate treatment locally and minimize systemic side effects. The preclinical investigations of NFMS discussed in the previous section reflect a broader trend in medical research that moves beyond their traditional role in controlled drug release systems. The advancements in conventional microsphere technology could serve as a model for the future development of NFMS, suggesting a promising outlook for NFMS in clinical trials as the field progresses.

Securing robust intellectual property (IP) protection is crucial to safeguard proprietary technologies and innovations, yet it often presents challenges such as potential infringement concerns or complexities in navigating existing patent landscapes.^176^ To address these challenges, a comprehensive IP strategy is essential. This involves conducting thorough prior art searches to identify existing patents and ensuring that NFMS innovations are novel and nonobvious. Filing strategic patent applications early in the development process provides a foundation for IP protection. Collaborations and partnerships with research institutions or industry leaders can also strengthen IP positions, fostering a mutually beneficial environment for knowledge exchange while protecting against IP-related disputes. Additionally, monitoring the IP landscape for emerging technologies and potential infringements allows for timely adjustments to the IP strategy.^177,178^

Lastly, addressing ethical considerations in the development and regulatory approval of NFMS is integral to responsible research and patient care. One ethical consideration involves informed consent procedures, ensuring that participants fully understand the nature of the study, potential risks, and benefits before enrolling in clinical trials.^179^ Transparent communication, patient education materials, and thorough discussions with participants contribute to obtaining informed and voluntary consent.^180^ Another ethical consideration is the use of animal models in preclinical studies. Researchers must prioritize the humane treatment of animals, minimize pain and distress, and adhere to established ethical guidelines for animal research. Alternative methods, such as in vitro models or computational simulations, can be explored to reduce reliance on animal testing.^181^

## 7 Concluding Remarks and Future Directions

In conclusion, NFMS represent a promising biomimetic platform for advancing the field of bone tissue regeneration. This review has highlighted the versatility and potential of NFMS in providing tailored solutions for addressing the complex challenges associated with bone repair and regeneration. Through meticulous design, integration of biomimetic cues, and precise control over scaffold properties, NFMS offer enhanced bioactivity, mechanical support, and therapeutic delivery capabilities, making them highly suitable for promoting osteogenesis and facilitating bone tissue healing. Nevertheless, considerable effort is still required before functional NFMS can be effectively applied in clinical practice.

One of the first challenges that needs to be addressed is that of scalability in NFMS production. For this, several strategies can be explored. First, optimizing fabrication techniques to enable large-scale production while maintaining reproducibility and quality is essential. This may involve automating processes or implementing continuous manufacturing methods.^182^ Additionally, advancing biomaterials engineering to develop cost-effective and readily available materials for NFMS fabrication can enhance scalability. Future work in this domain can focus on exploring microfluidic-based fabrication platforms that can facilitate high throughput production of NFMS with uniform size, morphology, and composition.^183^ By streamlining manufacturing processes and optimizing resource utilization, economies of scale can be achieved, leading to lower per-unit costs. This emphasis on cost-effective production methods ensures that NFMS-based therapies are affordable and widely available, ultimately benefiting patients in need of bone tissue regeneration treatments.

Another important challenge in commercialization is the stability of NFMS. Currently, there is a lack of preclinical studies focused on evaluating the long-term stability of entrapped biomolecules within NFMS, which is essential for ensuring their efficacy and bioactivity over extended periods. Additionally, there is a need to assess NFMS’s performance after exposure to different storage conditions that they may encounter if commercialized, such as variations in temperature and humidity.^184^ To enhance the stability of NFMS-based products, researchers should focus on developing formulations with prolonged shelf life, reducing the reliance on stringent cold storage conditions. Freeze-drying is a promising technique that can be explored to achieve this goal, as it preserves the functionality of biomolecules and allows for the production of stable, lyophilized products that are less susceptible to degradation during storage and transportation.^185^

Although NFMS presents substantial benefits in bone tissue engineering, it is crucial to also consider the advantages of other scaffold types. This comparison is essential for identifying and targeting areas where further improvements in NFMS can be achieved. To begin with, the mechanical properties of NFMS, while superior to many biomaterial-based platforms, may not match the robustness of traditional, rigid scaffolds (such as those made from metal or ceramic materials).^186^ NFMS typically exhibit lower compressive and tensile strengths due to their polymeric and fibrous nature, which can limit their use in high load-bearing applications where high mechanical integrity is crucial.^187,188^ Second, the microscale size of NFMS can pose challenges in achieving adequate vascularization and integration with host tissue, especially in large defects.^189^ The size and porosity of NFMS, though ideal for cellular interactions and nutrient diffusion at the microscale, may not provide sufficient macroscale architecture necessary to support new tissue growth and vascular network formation across larger spans of bone defects.^190^ Also, it is challenging to control the degradation rate of NFMS to match the rate of new bone formation. If the NFMS degrade too quickly, they may fail to provide sufficient support during the critical early stages of healing; if too slow, they might hinder the integration of new bone tissue.^191^

Injectable hydrogels represent a compelling alternative to NFMS due to their minimally invasive application that minimizes recovery time and associated complications. The viscoelastic properties of hydrogels provide a cushioning effect, which can be advantageous in protecting newly forming tissue from mechanical stress while still allowing sufficient mechanical signals necessary for bone growth and remodeling.^192,193^ While NFMS provide structural support, they are generally more rigid and less capable of mimicking the viscoelastic properties of natural tissues. Incorporating viscoelastic elements into NFMS could enhance their biomechanical compatibility and support more natural bone tissue formation.^194,195^ Nanomaterial-based scaffolds, such as those incorporating carbon nanotubes, graphene, or bioactive ceramics like hydroxyapatite, have demonstrated exceptional mechanical strength and osteoconductivity properties.^196^ These materials can significantly enhance the mechanical robustness of scaffolds, making them more suitable for load-bearing applications, which is a current limitation of NFMS. Furthermore, nanomaterials’ high surface area-to-volume ratio allows for a more efficient loading and sustained release of therapeutic agents.^197^ The electrical properties of certain nanomaterials, like carbon nanotubes and graphene, can be leveraged to promote cellular activities through electrical stimulation, a feature that is not typically inherent to NFMS.^198^ This aspect could be particularly beneficial in regenerative strategies where electrical cues are known to influence cell behavior and tissue formation.

An interesting area that can be explored is the integration of personalized and patient-specific approaches to tailor NFMS-based therapies to the specific anatomical and physiological characteristics of individual patients. This customization begins with the utilization of advanced imaging modalities (such as CT or MRI) to obtain high-resolution 3D reconstructions of the patient’s bone defects.^199^ These imaging data provide invaluable insights into the size, shape, location, and surrounding tissue architecture of the defect. Based on these data, additive manufacturing technologies such as 3D printing can be leveraged, wherein NFMS can possibly be integrated as part of the “bio-ink” that forms patient-specific NFMS-integrated scaffolds.^200,201^ As it would be designed to closely match the dimensions and contours of the patient’s bone defect, it would promote optimal fit and contact with the surrounding tissue for improved integration and functionality.^202,203^ Bioactive factors can be incorporated into the NFMS matrix using spatially controlled deposition methods, allowing for the creation of gradient or multilayered scaffolds that mimic the native tissue microenvironment and promote enhanced tissue regeneration.^204^ Moreover, personalized NFMS-based therapies can harness patient-derived cells and bioactive factors to further augment bone healing outcomes. MSCs or osteoprogenitor cells harvested from the patient’s bone marrow or adipose tissue can be integrated into NFMS scaffolds to enhance osteogenic differentiation and tissue regeneration.^205^ The composition and concentration of bioactive factors encapsulated within NFMS can be customized based on the patient’s specific bone healing profile and therapeutic requirements. This ensures precise modulation of the regenerative microenvironment.^206^

Ultimately, the clinical translation of personalized NFMS-based therapies requires rigorous evaluation of safety, efficacy, and long-term outcomes through preclinical studies and clinical trials. Predictive models and computational simulations integrating patient-specific data on bone biomechanics, physiology, and genetic predisposition can aid in treatment planning and outcome prediction, guiding clinical decision-making and optimizing personalized NFMS design.^207^ Standardized protocols and guidelines for patient screening, treatment selection, surgical procedures, and postoperative care are essential to ensure consistency and reproducibility across different clinical settings, paving the way for the widespread adoption of personalized NFMS-based approaches in bone tissue regeneration.^208,209^ With ongoing research efforts, interdisciplinary collaborations, and advancements in biomaterials science and tissue engineering, NFMS-based approaches hold great promise for translating innovative therapies from the laboratory to the clinic.
