# Correction to “Nanofibrous Microspheres: A Biomimetic Platform for Bone Tissue Regeneration”

**DOI:** 10.1021/acsabm.4c01057

**Published:** 2024-08-20

**Authors:** Nimeet Desai, Shreya Pande, Lalitkumar Vora, Nagavendra Kommineni

In the published version of our review paper, we unintentionally omitted the citation for Figure 1(A). This panel was recreated from a figure published in an open access (CC BY 4.0) review article by de Wildt et al. (10.1016/j.cobme.2019.05.005). We would like to correct the figure caption to properly attribute the original source. The corrected caption should read as follows:

Figure 1. (A) Assembly of collagen from its fundamental structures to a complex network that forms the ECM within the bone. The organic matrix of bone is characterized by organized collagen fibrils at the nanometer scale and a densely aligned collagen fiber network at the micrometer scale. **Reproduced from** ref (17)**, 2019, Elsevier, Inc. This work is licensed under a CC BY 4.0 license.** (B) Second harmonic generation image depicting a densely packed and aligned collagen fiber network in human femoral cortical bone. This image vividly captures the robust and organized texture of collagen fibers within the network, highlighting their alignment and density, essential for the mechanical strength of bone (scale bar: 200 μm). Reproduced with permission from ref (18). Copyright Springer Nature 2017. (C) Electron microscopy image of cortical bone collagen fibrils. This high-resolution image provides a detailed view of the parallel alignment of individual collagen fibrils, underscoring the precision of molecular organization at the smallest scale within bone ECM. Reproduced with permission from ref (19). Copyright Elsevier 2000.

Due to the addition of this new citation, the numbering of subsequent citations in the text has been adjusted accordingly. The original citation numbers of refs (1−16) are the same. References (17−209) are now refs (18−210). The reference list has been updated here to reflect these changes. The review’s findings and content are unaffected by this change.
